# Regulation of endometrial cancer cell growth by luteinizing hormone (LH) and follicle stimulating hormone (FSH)

**DOI:** 10.1054/bjoc.2000.1507

**Published:** 2000-12

**Authors:** S Davies, C M R Bax, E Chatzaki, T Chard, R K Iles

**Affiliations:** Williamson Laboratory, East Wing, St Bartholomew's Hospital, West Smithfield, London, EC1A 7BE, UK; Department of Pharmacology, Medical School, University of Crete, Heraklion, 71110, Greece; Reproductive Physiology, St Bartholomew's Hospital, St Bartholomew's Close, West Smithfield, London, EC1A 7BE, UK

**Keywords:** endometrial cancer, luteinizing hormone (LH), follicle stimulating hormone (FSH), GnRH analogues

## Abstract

Gonadotrophin releasing hormone analogues (GnRHa) have been used to treat recurrent endometrial cancer. However, the mode of action is uncertain. Our previous studies showed no direct effect of GnRHa on endometrial cancer cell growth in vitro. We have now examined the effect of luteinizing hormone (LH) and follicle stimulating hormone (FSH) on endometrial cancer cell growth. The aim was to determine whether suppression of pituitary LH and FSH by GnRHa could explain the tumour regression seen in up to 44% of patients treated with this drug. We show that recombinant human LH and FSH (rhLH and rhFSH) produce a concentration dependent stimulation of the endometrial cancer cell line HEC-1A, in serum-free medium (maximum increase of 62 and 50% respectively relative to untreated controls). This increase is equivalent to that obtained by addition of 10% newborn calf serum. Growth of the Ishikawa cell line in culture increases in the presence of rhLH (maximum increase of 67%) but not with rhFSH. Using RT-PCR, we show that the Ishikawa cell line intermittently expresses receptor mRNA of LH but not of FSH; there is no expression of either mRNA by HEC-1A. Classically, both LH and FSH act via cAMP linked membrane receptors. However, neither rhLH nor rhFSH elicit cAMP production in either of our endometrial cancer cell lines. Thus, although a growth response to LH and FSH can be shown, and some cells express the LH receptor, stimulation appears to be via a pathway separate from that of the classical gonadotrophin receptor. © 2000 Cancer Research Campaign http://www.bjcancer.com


*British Journal of Cancer* (2000) **83**(12), 1730–1734
© 2000 Cancer Research Campaign
doi: 10.1054/ bjoc.2000.1507, available online at http://www.idealibrary.com on IDEAL®

### Regulation of endometrial cancer cell growth by luteinizing hormone (LH) and follicle stimulating hormone (FSH)

**S Davies[1], CMR Bax[1], E Chatzaki[2], T Chard[3] and RK Iles[1]**

[1]Williamson Laboratory, East Wing, St Bartholomew's Hospital, West Smithfield, London EC1A 7BE, UK; [2]Department of Pharmacology, Medical School, University of Crete, Heraklion 71110, Greece; [3]Reproductive Physiology, St Bartholomew's Close, St Bartholomew's Hospital, West Smithfield, London EC1A 7BE, UK

**Summary** Gonadotrophin releasing hormone analogues (GnRHa) have been used to treat recurrent endometrial cancer. However, the mode of action is uncertain. Our previous studies showed no direct effect of GnRHa on endometrial cancer cell growth in vitro. We have now examined the effect of luteinizing hormone (LH) and follicle stimulating hormone (FSH) on endometrial cancer cell growth. The aim was to determine whether suppression of pituitary LH and FSH by GnRHa could explain the tumour regression seen in up to 44% of patients treated with this drug. We show that recombinant human LH and FSH (rhLH and rhFSH) produce a concentration dependent stimulation of the endometrial cancer cell line HEC-1A, in serum-free medium (maximum increase of 62 and 50% respectively relative to untreated controls). This increase is equivalent to that obtained by addition of 10% newborn calf serum. Growth of the Ishikawa cell line in culture increases in the presence of rhLH (maximum increase of 67%) but not with rhFSH. Using RT-PCR, we show that the Ishikawa cell line intermittently expresses receptor mRNA of LH but not of FSH; there is no expression of either mRNA by HEC-1A. Classically, both LH and FSH act via cAMP linked membrane receptors. However, neither rhLH nor rhFSH elicit cAMP production in either of our endometrial cancer cell lines. Thus, although a growth response to LH and FSH can be shown, and some cells express the LH receptor, stimulation appears to be via a pathway separate from that of the classical gonadotrophin receptor. © 2000 Cancer Research Campaign http://www.bjcancer.com

**Keywords:** endometrial cancer; luteinizing hormone (LH); follicle stimulating hormone (FSH); GnRH analogues

Endometrial cancer occurs most commonly in postmenopausal women and is therefore coincidentally associated with elevated plasma LH and FSH levels (Nagamani et al, 1993). LH and FSH secretion is partly controlled by hypothalamic gonadotrophin releasing hormone (GnRH) and it is notable that recurrent endometrial cancer can be treated with GnRH analogues (Gallagher et al, 1991). However, the exact mechanism by which these analogues exert their clinical effect on endometrial cancer cell growth is not fully understood. The in vivo physiological response to GnRH administration is an initial rise followed by suppression of LH and FSH secretion (Dowsett et al, 1988). Emons et al (1993) found two types of GnRH binding sites in the HEC-1A and Ishikawa endometrial cancer cell lines; specific high affinity GnRH receptors were also found in the DU-145 prostate tumours (Lamharzi et al, 1998). However, we found no functional GnRH receptor in normal or malignant endometrium and no direct growth modulating effect in vitro on endometrial cancer cells (Chatzaki et al, 1996).

In this study we explore the hypothesis that the antitumour activity of GnRH is an indirect consequence of reducing circulating LH and FSH levels. We describe the growth response of endometrial cancer cells to administration of LH and FSH; the second messenger response and the expression of LH and FSH receptors by these cells.

*Received 15 September 1999*
*Revised 19 July 2000*
*Accepted 15 August 2000*

*Correspondence to:* RK Iles

### MATERIALS AND METHODS

### Cell lines

The human endometrial adenocarcinoma cell line Ishikawa (Nishida et al, 1985) was a gift from Dr J White (Department of Reproductive Physiology, Hammersmith Hospital, London, UK) and the human endometrial papillary adenocarcinoma cell line HEC-1A was obtained from the American Type Culture Collection (Bethesda, MD). The human embryonic kidney cell lines 293L and 293F derived from 293 cells after stable transfection with LH and FSH receptor cDNA respectively were donated by Dr D Segaloff (University of Iowa College of Medicine, Iowa, USA).

### LH and FSH

Pituitary-derived LH and FSH were obtained from Sigma (Dorset, UK) and recombinant human LH and FSH were obtained from Dr M Page (Serono).

### Growth experiments

293, 293L and 293F cells were grown in phenol-red free DMEM media (Sigma-Aldrich Company Ltd, Dorset, UK) supplemented with 5% heat-inactivated newborn calf serum (NCS), gentamycin, 10 mM HEPES, 2 mM glutamine plus antibiotic/antimycotic solution (Sigma, Dorset, UK). Ishikawa cells were grown in phenol-red free DMEM nutrient mixture F-12 Ham media (Sigma, Dorset,

UK) supplemented with 10% NCS, glutamine plus antibiotic/antimycotic solution (Sigma, Dorset, UK). The HEC-1A cells were grown in phenol-red free RPMI-1640 media (Sigma, Dorset, UK) supplemented with 10% NCS plus antibiotic/antimycotic solution (Sigma, Dorset, UK). All the cells were grown in 75 $cm^2$ flasks in a humidified atmosphere of 5% $CO_2$ at 37˚C.

For the growth experiments, cells were plated in 24-well microtitre plates with serum free media containing 0.1% bovine serum albumin ($10^4$ cells $well^{-1}$). After a 24-hour settling period, individual wells were treated with varying concentrations of pituitary derived or recombinant human LH and FSH. The cells were incubated for 6 days with a medium change every 2 days. The medium was then removed and the cells were lysed in 1 × Standard Saline Citrate/0.06% Sodium Dodecyl Sulphate overnight at –20˚C. DNA content was quantified by the method of Labarca and Paigen (1980).

### Cyclic AMP competitive binding assay

Total cAMP levels were measured after cell incubation for 15 min with increasing concentrations of LH and FSH (300–10 000 U $l^{-1}$) and forskolin (10 µM) in the presence of 0.25 mM 3-isobutyl-1-methylxanthine (Sigma, Dorset, UK). The cells were lysed by boiling, and the cell lysates were assayed for cAMP using a competitive binding assay. Briefly, this assay relies upon competition between [$^3$H] labelled cAMP (Amersham, Buckinghamshire, UK) and unlabelled cAMP in the sample for a crude cAMP binding protein prepared from bovine adrenal glands (Brown et al, 1971; Farndale et al, 1992). After incubation, free [$^3$H] cAMP is adsorbed onto charcoal and removed by centrifugation. Bound [$^3$H] cAMP remains in the supernatant and is measured by liquid scintillation counting. The protein content of cells was quantified using the Folin-Lowry method (Lowry et al, 1951) in order to standardize the cAMP responses.

### Extraction of messenger RNA

Messenger RNA was extracted using a standard isolation kit (Sigma, Dorset, UK). The cells were thawed, removed from medium, washed in sterile PBS, then lysed, homogenized and incubated at 45˚C before precipitation of the DNA with 5M $NaCl_{(aq)}$. The DNA was sheared and oligo (dT) cellulose was added to bind the mRNA. The oligo (dT) cellulose was then washed several times in binding buffer and low salt wash buffer before release of mRNA from the oligo (dT) cellulose using elution buffer. The final product was approximately, 0.05 ml of a solution containing 0.67–5.1 g $L^{-1}$ mRNA. This was stored at –70˚C.

### Reverse transcription-polymerase chain reaction (RT-PCR)

Messenger RNA (3–25.5 µg) was reverse transcribed using the oligo (dT) primer and murine leukaemia virus reverse transcriptase (MoMLV). Transcription was carried out in a volume of 30 µl containing 1× reverse transcription buffer, 0.075 mM dNTPs, 10 ng $\mu l^{-1}$ oligo $(dT)_{15}$ primer; 2 mM dithiothreitol (DTT) and 4 U $\mu l^{-1}$ MoMLV-reverse transcriptase. The reaction mixture was incubated at 42˚C for 1 h in a Techne thermal cycler.

5 µl of this cDNA preparation was subjected to 35 cycles of amplification using a Techne thermal cycler. The polymerase chain reactions (PCRs) were carried out in a 25 µl reaction mixture with a PCR bead (Amersham, Buckinghamshire, UK) containing 10 mM Tris-HCl (pH 9.0), 50 mM KCl, 1.5 mM $MgCl_2$, 200 µM dNTPs and 1.5 U of Taq DNA polymerase and 400 mM of each primer. A PCR was set up for the LH/FSH receptors using oligonucleotide primers that span intronic sequences specific for each receptor (Table 1).

After initial denaturation for 2 min at 94˚C, 35 cycles of amplification were performed with 1 min denaturation at 94˚C, 50 s annealing temperature and 50 s extension at 73˚C. The last cycle had an elongation time of 10 min at 73˚C.

### Gel electrophoresis

An aliquot of the PCR reaction mixture was examined on a 2% agarose gel, stained with ethidium bromide and photographed under UV light.

### Actin controls

In order to check the integrity of the reverse transcriptase reaction the expression of the house keeping gene actin was used as a control. A forward (CAG CCA TGT ACG TTG CTA TCC AGG) and reverse primer (TTG CGG ATG TCC ACG TCA CAC TTC) spanning intron 4 of the human cytoplasmic beta-actin gene (Nakajima-Iijima et al, 1985) were used in a PCR reaction as described above but with an annealing temperature of 66˚C. Genomic DNA contamination would yield a product of 579 bp and cDNA a product of 483 bp.

### RESULTS

### Stimulation of cell growth by LH and FSH

HEC-1A cell growth was stimulated by pituitary-derived LH and FSH by 75 and 77% respectively relative to untreated controls.

**Table 1** Primers used in PCR amplification for the LH and FSH receptors. The site of hybridization and annealing temperatures are shown

| Primer | Location | Nucleotide sequence | Annealing temperature |
|---|---|---|---|
| hLH-R 5′ | 676/698 | 5′-CCTGGATATTTCTTCCACCAAA-3′ | 55˚C |
| hLH-R 3′ | 1270/1291 | 5′-TGGCATGGTTATAGTACTGGC-3′ | 55˚C |
| hFSH-R 5′ | 264/285 | 5′-GGTGCATTTTCAGGATTTGGGG-3′ | 62˚C |
| hFSH-R 3′ | 527/552 | 5′-TTGTGTGGATGTTTATGTTATCTTG-3′ | 62˚C |

© 2000 Cancer Research Campaign

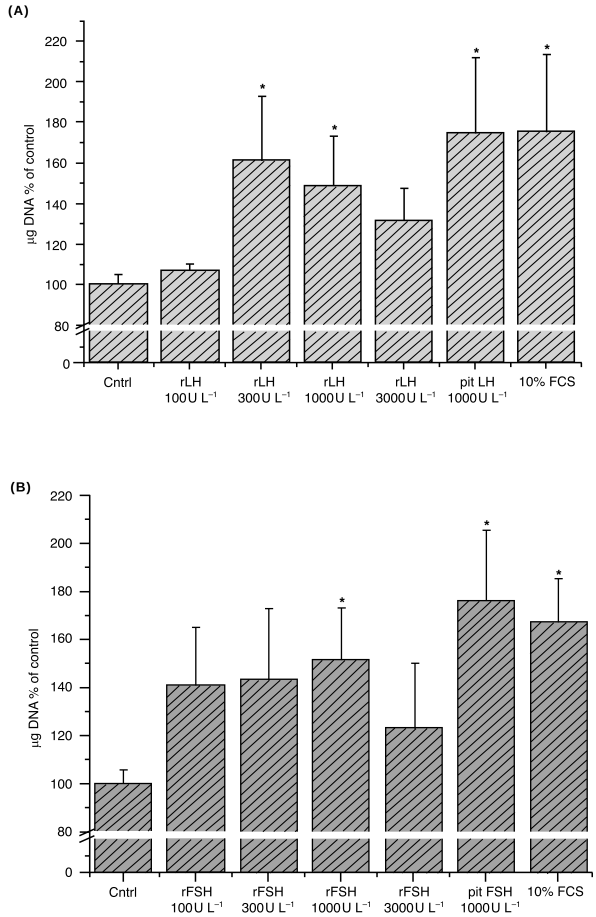

**Figure 1** Effect of varying concentrations of rhLH (**A**) and rhFSH (**B**) on the growth of endometrial cancer cell line HEC-1A in phenol red-free media over 6 days of treatment. Cells grown in 10% NCS were used as positive controls. All results are expressed as a percentage of non-treated controls. Each determination was performed in 15 replicates; bars represent standard error of the mean. The asterisk shows statistical significance ($P < 0.05$)

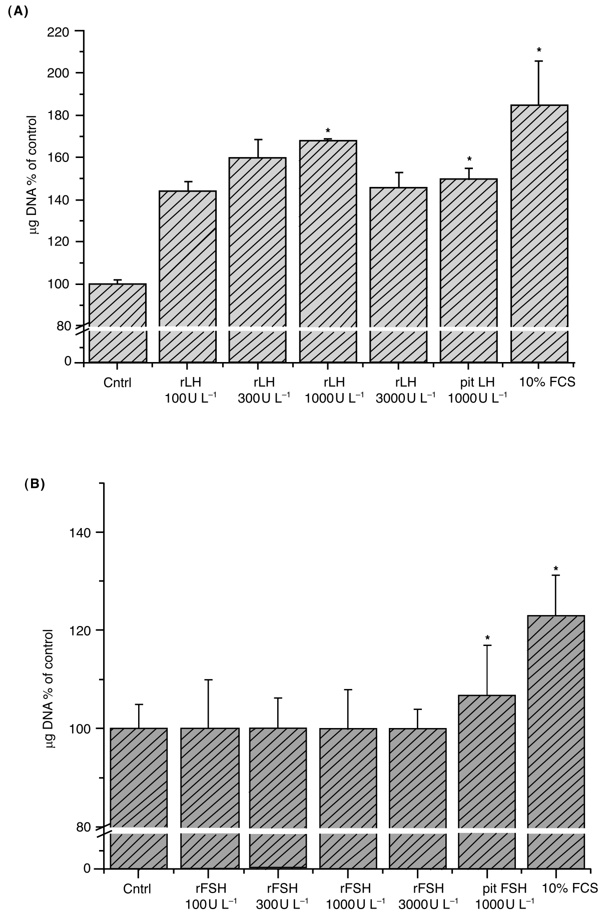

**Figure 2** Effect of varying concentrations of rhLH (**A**) and rhFSH (**B**) on the growth of endometrial cancer cell line Ishikawa in phenol red-free media over 6 days of treatment. Cells grown in 10% NCS were used as positive controls. All results are expressed as a percentage of non-treated controls. Each determination was performed in 15 replicates, bars represent Standard Error. The asterisk shows statistical significance ($P < 0.05$)

HEC-1A cell growth also increased in a concentration dependent manner when exposed to rhLH (100–300 U $L^{-1}$) and rhFSH (300–1000 U $L^{-1}$) with maximum increases of 62% and 50%, respectively. This is comparable to the effect of 10% NCS, in the presence of which cell numbers were stimulated relative to untreated controls by 76% and 68% (Figure 1).

The growth of Ishikawa cells was stimulated by pituitary-derived LH by 50%. Ishikawa cells also increased in culture in a dose-dependent manner when exposed to rhLH with a maximum increase of 67% at 1000 U $L^{-1}$. This is comparable to the effect of 10% NCS which stimulated an 85% increase (Figure 2). However, no growth effect was seen with rhFSH.

### Second messenger activity

Control cell lines 293L and 293F show a rise in cAMP production following stimulation with varying concentrations of LH and FSH respectively (Figure 3). By contrast, cAMP production did not increase in response to LH and FSH in either endometrial cancer cell line. All cell lines showed a dramatic increase in cytoplasmic cAMP (> 10 000 U $L^{-1}$) when exposed to 10 µM forskolin (results not shown).

### Expression of hLH receptor

The positive control yielded the expected size band (616 bp). The hLH receptor was expressed by the endometrial cancer cell line Ishikawa but not by HEC-1A (Figure 4). However, this band was not consistently seen in repeat experiments.

### Expression of the hFSH receptor

The positive control yielded the expected size band (289 bp). The hFSH receptor was not expressed by HEC-1A or Ishikawa (Figure 5).

### DISCUSSION

Endometrial cancer responds to treatment with GnRHa. Some 28% of postmenopausal women with recurrent endometrial cancer showed clinical improvement with monthly depot injections of GnRH analogues; long-term survival was 44% (Jeyarajah et al, 1996). The mechanism of this action is not understood. It has been suggested that GnRH analogues have a direct antitumour effect on endometrial cancer cells but there is no direct effect of GnRH analogues on endometrial cancer cell growth in vitro (Chatzaki

*© 2000 Cancer Research Campaign*

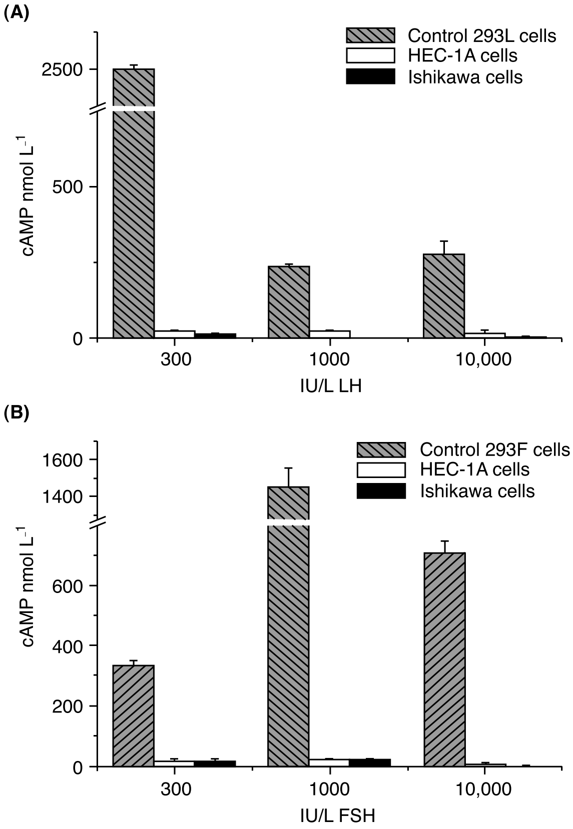

**Figure 3** (**A**) Cyclic AMP production by the gonadotrophin receptor-positive 293 cells and the endometrial cancer cell lines HEC-1A and Ishikawa in response to varying concentrations of LH (300–10 000 U $L^{-1}$). (**B**) Cyclic AMP production by the gonadotrophin receptor-positive 293 cells and the endometrial cancer cell lines HEC-1A and Ishikawa in response to varying concentrations of FSH (300–10 000 U $L^{-1}$)

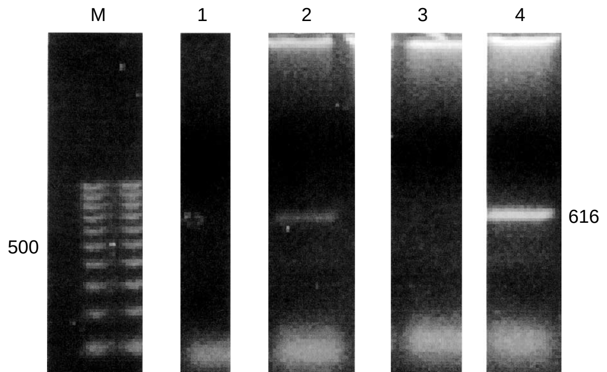

**Figure 4** PCR amplification of the hLH receptor. The PCR products were analysed by agarose gel electrophoresis and visualized by ethidium bromide staining under UV light. *M*, 100 bp DNA ladder. *Lane 1* derived from a PCR reaction in the absence of DNA. The PCR products from testis cDNA (*Lane 2*), HEC-1A cDNA (*Lane 3*) and Ishikawa cDNA (*Lane 4*) are presented. The predicted 616 bp band can be seen in the endometrial cancer cDNA for Ishikawa but not for HEC-1A

et al, 1996). Imai et al (1994) described GnRH receptor mRNA expression in the human endometrium but this could not be confirmed by other studies on normal or malignant endometrium (Chatzaki et al, 1996).

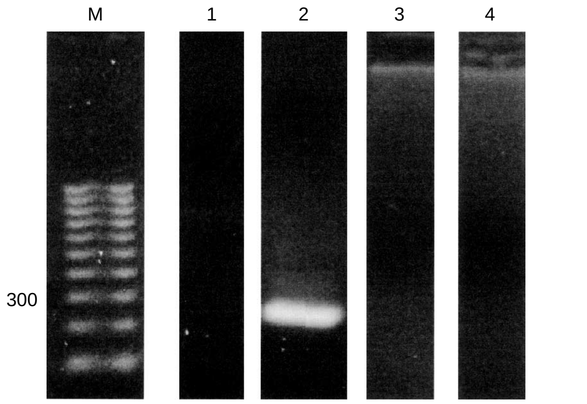

**Figure 5** PCR amplification of the hFSH receptor. The PCR products were analysed by agarose gel electrophoresis and visualized by ethidium bromide staining under UV light. *M*, 100 bp DNA ladder. *Lane 1* derived from PCR reaction in the absence of DNA. The PCR products from testis cDNA (*Lane 2*), HEC-1A cDNA (*Lane 3*) and Ishikawa cDNA (*Lane 4*) are presented. The predicted 289 bp size band cannot be detected in the endometrial cancer cDNA for HEC-1A or Ishikawa

GnRH analogues are widely used in cancer treatment: for example, they have been administered to postmenopausal women with locally advanced or metastatic breast cancer. The most notable endocrine change found in postmenopausal women treated with such analogues is a reduction in plasma LH and FSH concentrations. Dowsett et al (1988) reported that the mean serum LH level fell progressively to 8.2% of the pre-treatment value after 4 weeks of treatment and thereafter ranged between 5.5–6.2% of the pre-treatment level; the mean serum FSH level fell to 8.6% of the pre-treatment value after 3 weeks and thereafter ranged between 7.5–9.5% of the pre-treatment value. Similarly, in endometrial cancer patients treated with a GnRH analogue, gonadotrophin levels were suppressed within the first 2 months of use (Jeyarajah et al, 1996).

Given that there is no functional GnRH receptor in normal or malignant endometrium (Chatzaki et al, 1996), it was reasonable to assume that endometrial cancer may be associated with gonadotrophin receptor expression. Growth may be retarded by the profound and sustained suppression of the high postmenopausal gonadotrophin levels which occurs with GnRH analogue treatment. In this study, we show that growth of HEC-1A was stimulated by the addition of recombinant LH and to a lesser extent by FSH (Figure 1). Ishikawa cells also increased in culture in a dose-dependent manner when exposed to rhLH but not rhFSH (Figure 2). Simon et al (1983) have demonstrated that LH and FSH stimulated the growth of cell lines derived from malignant epithelial tumours and thus this study is in agreement. However, they went on to demonstrate gonadotrophin receptor mRNA expression by benign and malignant human epithelial lesions. However, at best we were able to demonstrate an intermittent expression of LH receptor but not FSH receptor mRNA (Figures 4 and 5). Nevertheless, Lin et al (1994) have also demonstrated LH receptor mRNA expression by endometrial cancer cell lines.

Despite this, it is not clear how gonadotrophin receptor activation can regulate cell growth. LH and FSH normally act via cAMP. In this study, these hormones did not elicit cAMP production in HEC-1A or Ishikawa cells (Figure 3) although they did stimulate cell growth (Figures 1 and 2). Though we were unable to show a classical cAMP second messenger response to LH and FSH, it is

© 2000 Cancer Research Campaign possible that they might act via a different pathway to promote cell growth in endometrial cancer. Gonadotrophin receptors are also coupled to the inositol phosphate-protein kinase C (IP-PKC) pathway (Grudermann et al, 1992). However, gonadotrophin levels sufficient for $IP_3$ stimulation are observed only during the pre-ovulatory surges and pregnancy, suggesting that the physiological role, if any, of the IP-PKC pathway in conveying the biological actions of gonadotrophins to target cells is limited to these situations (Grudermann et al, 1992). These studies strongly suggest that high postmenopausal levels of LH and possibly FSH are stimulating endometrial cancer cell growth but not via the classical LH receptor-cAMP pathway.

### ACKNOWLEDGEMENTS

We are most grateful to Dr Chris Gallagher for his help in the initial part of this study. We also thank Dr M Page of Serono Labs for supplying us with recombinant LH and FSH. This work was supported by the Barts Cancer Research Committee. Dr Chris Bax acknowledges the support of Barts Foundation for Research and the Joint Research Board. Miss Suzy Davies is a recipient of a Chard PhD Studentship.

### REFERENCES

Brown BL, Albano JD, Ekins RP and Sgherzi AM (1971) A simple and sensitive saturation assay method for the measurement of adenosine 3′:5′-cyclic monophosphate. *Biochem J* **121(3)**: 561–562

Chatzaki E, Bax CMR, Eidne KA, Anderson L, Grudzinskas JG and Gallagher CJ (1996) The expression of gonadotrophin releasing hormone and its receptor in endometrial cancer and its relevance as an autocrine growth factor. *Cancer Res* **56**: 2059–2065

Dirnhofer S, Berger C, Hermann M, Steiner G, Madersbacher S and Berger P (1998) Coexpression of gonadotrophic hormones and their corresponding FSH and LH/CG receptors in the human prostate. *The Prostate* **35**: 212–220

Dowsett M, Cantwell B, Anshumala L, Jeffocate SL and Harris AL (1988) Suppression of postmenopausal ovarian steroidogenesis with the luteinising hormone-releasing hormone agonist goserelin. *J Clin Endocrinol Metabolism* **66**: 672–677

Emons G, Schroder B, Ortmann O, Westphalen S, Schulz KD and Schally AV (1993) High affinity binding and direct antiproliferative effects of luteinising hormone-releasing hormone analogs in human endometrial cancer cell lines. *J Clin Endocrinol Metabolism* **77(6)**: 1458–1464

Farndale RW, Allan LM and Martin BR (1992) Adenylate cyclase and cAMP (Chapter 4). In: Milligan G (ed), *Signal Transduction: A Practical Approach.* OUP, Oxford

Gallagher CJ, Oliver RTD, Oram DH, Fowler CG, Blake PR, Mantell BS, Slevin ML and Issacs NW (1991) A new treatment for endometrial cancer with gonadotrophin releasing hormone analogue. *Br J Obst Gynaecol* **98**: 1037–1041

Grudermann T, Birnbaumer M and Birnbaumer L (1992) Evidence for dual coupling of the murine luteinising hormone receptor to adenylate cyclase and phosphoinositol breakdown and $Ca^{2+}$ mobilisation. *J Biol Chem* **267**: 4479–4488

Imai A, Ohno T, Iida K, Fuseya T, Furui T and Tamaya T (1994) Presence of gonadotrophin-releasing hormone receptor and its messenger ribonucleic acid in endometrial carcinoma and endometrium. *Gynaecol Oncol* **55(1)**: 144–148

Jeyarajah AR, Gallagher CJ, Blake PR, Oram DH, Dowsett M, Fisher C and Oliver RTD (1996). Long term follow-up of Gonadotrophin-releasing hormone analog treatment for recurrent endometrial cancer. *Gyn Oncol* **63**: 47–52

Labarca C and Paigen K (1980) A simple and sensitive DNA assay procedure. *Analytical Biochemistry* **102**: 344–352

Lamharzi N, Halmos G, Jungwirth A and Schally AV (1998) Decrease in the level and mRNA expression of LH-RH and EGF receptors after treatment with LH-RH antagonist cetroelix in DU-145 prostate tumour xenografts in nude mice. *Int J Oncol* **13(3)**: 429–435

Lin J, Lei ZM, Lojun S, Rao CV, Satyaswaroop PG and Day TG (1994) Increased expression of luteinising hormone/human chorionic gonadotrophin receptor gene in human endometrial carcinomas. *J Clin Endocrinol Met* **79(5)**: 1483–1491

Lowry O, Rosebrough NJ, Farr AL et al (1951) Protein determination with the Folin phenol reagent. *J Biol Chem* **193**: 265–275

Nagamani M, Doherty MG, Smith ER and Chandrasekhar Y (1993) Increased bioactive luteinising hormone levels in postmenopausal women with endometrial cancer. *Am J Obst Gynaecol* **167**: 1825–1830

Nakajima-Iijima S, Hamada H, Reddy P and Kakunaga T (1985) Molecular structure of the human cytoplasmic beta-actin gene: interspecies homology of sequences in the introns. *Proc Natl Acad Sci USA* **82**: 6133–6137

Nishida M, Kasahara K, Kaneto M, Iwasaki H and Hayashi K (1985) Establishment of a new human adenocarcinoma cell line, Ishikawa cells, containing oestrogen and progesterone receptors. *Acta Obstetrics and Gynaecology Journal of Japan* **37**: 1103–1111

Simon WE, Albrecht M, Hansel M, Dietel M and Holzel F (1983) Cell lines derived from human ovarian carcinomas: growth stimulation by gonadotropic and steroid hormones. *J Natl Cancer Inst* **70(5)**: 839–845

 *© 2000 Cancer Research Campaign*
